# Long-term associative learning predicts verbal short-term memory performance

**DOI:** 10.3758/s13421-017-0759-3

**Published:** 2017-10-02

**Authors:** Gary Jones, Bill Macken

**Affiliations:** 1 Department of Psychology, Nottingham Trent University, 50 Shakespeare Street, Nottingham, NG1 4FQ UK; 2 School of Psychology, Cardiff University, Cardiff, UK

**Keywords:** Short-term memory, Associative learning, Digit span, Nonword repetition, CLASSIC

## Abstract

Studies using tests such as digit span and nonword repetition have implicated short-term memory across a range of developmental domains. Such tests ostensibly assess specialized processes for the short-term manipulation and maintenance of information that are often argued to enable long-term learning. However, there is considerable evidence for an influence of long-term linguistic learning on performance in short-term memory tasks that brings into question the role of a specialized short-term memory system separate from long-term knowledge. Using natural language corpora, we show experimentally and computationally that performance on three widely used measures of short-term memory (digit span, nonword repetition, and sentence recall) can be predicted from simple associative learning operating on the linguistic environment to which a typical child may have been exposed. The findings support the broad view that short-term verbal memory performance reflects the application of long-term language knowledge to the experimental setting.

Tests that assess the ability to process verbal information in the short term hold a central place in the investigation of the constituent processes that underlie the range of broader linguistic skills. These tests, such as digit span, nonword repetition, and sentence recall—what we might broadly refer to as verbal short-term memory (vSTM) tests—are routinely used not only as tools for investigating putative vSTM processes in themselves but also as tools for the investigation of linguistic and other higher level cognitive skills. On the basis of these tests, several decades of research has shown relationships involving vSTM across a wide range of domains in typical and atypical development, from reading and vocabulary to reasoning and problem solving (e.g., Albert & Steinberg, 2011; Doebel, Rowell, & Koenig, 2016; Gathercole, 2006; Rispens & Baker, 2012). Not only does the precise nature of these relationships remain controversial but there are also different views about what sort of constituent processes are actually being assessed in vSTM tasks.

One broad view is that the development of certain skills depends on the operation of systems that enable the short-term maintenance and manipulation of information in order to enable long-term learning (e.g., Baddeley, Gathercole, & Papagno, 1998; Page & Norris, 2009). Another approach sees performance on vSTM tasks being an outcome, rather than a cause, of other aspects of language development (e.g., MacDonald & Christiansen, 2002; Melby-Lervåg, Lyster, Klem, Hagtvet, & Hulme, 2012). For example, increases in vocabulary and its concomitant expansion of lexical phonology enables more ready processing of the novel phonological strings presented in nonword repetition tasks (G. Jones, 2016; Melby-Lervåg et al., 2012; Messer, Verhagen, Boom, Mayo, & Leseman, 2015).

We present an alternative view that accords with the view of vSTM being an outcome rather than a cause of language development. However, rather than being domain-specific, we argue that performance in vSTM measures primarily reflects domain-general associative learning operating on the linguistic experience of the rememberer. Associative learning has provided explanations for effects seen across a number of domains. For example, object labelling in infancy based on specific features (Rakison, Lupyan, Oakes, & Walker-Andrews, 2008) and on the basis of solid versus nonsolid characteristics (Colunga & Smith, 2005); the development of visual representations of scenes (Fiser & Aslin, 2002) and the development of imitation (Catmur, Walsh, & Heyes, 2009); the infant’s ability to segment continuous sound sequences into words (Aslin, Saffran, & Newport, 1998) and to subsequently link those sounds to meanings (Hay, Pelucchi, Estes, & Saffran, 2011); discovering patterns within visual stimuli (Kirkham, Slemmer, & Johnson, 2002); and discovering word-referent mappings (Smith & Yu, 2008). Clearly, our associative learning approach is not novel; yet it is one that has received little consideration within the vSTM literature, despite potentially being able to provide a parsimonious explanation for the effects seen in vSTM tasks.

Associative learning focuses on experience that relates to the task at hand, which for vSTM tasks equates to linguistic experience. There is now a considerable body of evidence implicating long-term language knowledge in performance in the short-term setting. This knowledge may relate to semantic properties of verbal material (e.g., Allen & Hulme, 2006; Walker & Hulme, 1999), the lexicality and frequency of occurrence of verbal items (e.g., Hulme, Maughan & Brown, 1991; Hulme et al., 1997), and the correspondence between the phonological structure of the items and that of participants’ native language (e.g., Gathercole, Frankish, Pickering, & Peaker, 1999; G. Jones, Tamburelli, Watson, Gobet, & Pine, 2010). While explanations of such effects typically invoke mechanisms for the more robust encoding or retrieval of the nominal items, as a function of how closely they correspond to the long-term linguistic repertoire of the participant, there is also evidence showing an influence of sequence-level factors that transcend the properties of the individual items themselves. For example, short-term serial recall performance is facilitated when item sequences are such that their coarticulatory transitions are relatively fluent or familiar than when they are not, even when the items making up those sequences are equivalently familiar (Murray & Jones, 2002; Woodward, Macken, & Jones, 2008). Prior, passive exposure to varying interitem transitional probabilities also leads to subsequent superior serial recall performance for sequences more closely matching those preexposed transitional probabilities (e.g., M. Botvinick, 2005; M. Botvinick & Bylsma, 2005; Majerus, Perez, & Oberauer, 2012), reflecting the impact of implicit learning of statistical regularities within sound sequences (e.g., Aslin et al., 1998) on performance in the short-term memory setting.

Here, we adopt a corpus-based approach in order to examine the way in which vSTM tasks that are typically used in developmental settings can be seen as reflecting long-term associative learning processes operating on the linguistic environment of the child. We do this by computationally modeling the linguistic knowledge that a typical child can be expected to have gained. Since vSTM tasks are typically concerned with the maintenance and/or reproduction of sequences of verbal information, the key associative learning aspect of linguistic knowledge that should apply relates to just such sequential knowledge. Our model therefore focuses on associative learning operating on the sequential properties of the linguistic input. Specifically, we use a set of corpora of child-directed language as a proxy for the linguistic experience of 6-year-old children and show how basic associative (sequential) learning operating on that experience predicts the pattern of performance on versions of digit span, nonword repetition, and sentence recall tasks. We begin by outlining a computational model of associative (sequential) learning as applied to the linguistic setting. Our broad theoretical orientation here is that performance in the vSTM setting is governed by domain-general associative (sequential) learning that enables the application of long-term linguistic knowledge and skill to the verbal material presented to the participant in that vSTM setting (D. M. Jones, Macken, & Nicholls, 2004; B. Macken, Taylor, & Jones, 2014; W. J. Macken & Jones, 2003). In broad terms, this means that the closer the vSTM material corresponds to the linguistic knowledge and experience of the rememberer, the better performance in the vSTM task will be.

Importantly, although the sequences presented to participants in vSTM settings are by design novel (i.e., obviously familiar sequences such as runs or acronyms are excluded), this “novelty” is always a matter of degree: some novel sequences will more closely match the linguistic experience of the rememberer than others. We show that this grading of novelty is at play within our computational model of associative learning within those vSTM tasks typically used in the developmental setting. We then show in two behavioral experiments that the efficiency which the model processes the types of material presented to children in vSTM tasks predicts how children perform in those settings, and we go on to discuss theoretical and methodological implications of these findings as they relate to the investigation of short-term memory and to domain-general mechanisms on the part of the rememberer.

## CLASSIC: a computational model of associative (sequence) learning

CLASSIC (Chunking Lexical and Sublexical Sequences In Children) is a computational model of sequence learning (G. Jones, 2016; G. Jones, Gobet, Freudenthal, Watson, & Pine, 2014; G. Jones & Macken, 2015) whereby incrementally larger chunks of information are learned based on the model’s increased exposure to sequential input. Since the language domain offers the opportunity to estimate the type of sequence knowledge that one may experience (e.g., via child-directed speech samples), CLASSIC has focused on the language domain in investigations of how domain-general mechanisms influence task performance. CLASSIC is therefore presented with naturalistic word-delimited child-directed speech converted into phonemes and learns sequences from the input in a bottom-up fashion.

The learning mechanism is simple: From a given utterance, recode the utterance into as few meaningful units (chunks) as possible, based on existing chunked knowledge, then learn a new chunk for each adjacent pair of chunks in the recoded utterance. While we refer to “chunks” in relation to the model’s learning, CLASSIC is serving as a proxy to the type of associative learning that may take place for a given input and the results from the model will therefore indicate the effect that associative learning may have on task performance. In this context associative learning might include increased perceptual sensitivity to sequences of sounds and words that appear often in the input and/or the improvement in articulatory motor coordination that arises from practice in producing those sound sequences.

CLASSIC begins with knowledge of the basic phonemes in standard British English (one chunk for each phoneme). Given this basic starting knowledge, the first phonemic utterance presented to CLASSIC will therefore be converted into individual phoneme chunks (e.g., the utterance “Daddy’s ball”, d æ d iː z / b ɔː l, would be recoded into eight chunks: d, æ, d, iː, z, b, ɔː, l). Learning will then create a new chunk for each pair of adjacent chunks without crossing word boundaries unless the chunks themselves are words or multiword sequences (dæ, æd, diː, iːz / bɔː, ɔːl for the example utterance). Should the same utterance be repeated, CLASSIC would now use the newly learned chunks to recode the utterance using fewer chunks than were required on first presentation (i.e., dæ, diː, z / bɔː, l). Learning would create new chunks for each of the adjacent recoded chunks (i.e., dædiː, diːz / bɔːl). Table 1 gives an example of how learning would progress when the same utterance exists four times in succession in the child-directed speech.

**Table 1:** Demonstration of how CLASSIC’s learning progresses when the utterance “Not that big” appears four times in the input. The examples show learning occurring at every opportunity (i.e., a learning rate of 1.00) whereas in reality the learning rate is set to .50

Utterance	Recoded utterance	Chunks learned
n ɒ t / ð æ t / b ɪ g	n, ɒ, t / ð, æ, t / b, ɪ, g	nɒ, ɒt / ðæ, æt / bɪ, ɪg
n ɒ t / ð æ t / b ɪ g	nɒ, t / ðæ, t / bɪ, g	nɒt / ðæt / bɪg
n ɒ t / ð æ t / b ɪ g	nɒt / ðæt / bɪg	nɒt ðæt, ðæt bɪg
n ɒ t / ð æ t / b ɪ g	nɒt ðæt, bɪg	nɒt ðæt bɪg

CLASSIC’s learning mechanism potentially means acquiring a great deal of knowledge on every presentation of an input utterance; however, in order that the model does not learn spurious sequences, the learning rate is set to .50 such that any sequence must be encountered twice on average in order to be learnt as a new chunk (n.b. previous work using CLASSIC set the learning rate to 1.00 because less input was used).

### Input to the model

Input to CLASSIC is a combination of maternal utterances directed at 2-year-old to 3-year-old children from the Manchester corpus (Theakston, Lieven, Pine, & Rowland, 2001) and similar input directed at 4-year-olds to 5-year-olds that is available on CHILDES (MacWhinney, 2000). As the children in our behavioral studies are 6 years of age, the input was supplemented by story books aimed at 4-year-old to 6-year-old children (e.g., *Alice in Wonderland*). The “younger” (2-year-old to 3-year-old) input contained more than 300,000 utterances across 12 different mothers, whereas the “older” (4-year-old to 6-year-old) input contained 75,981 utterances in total. A random sample of 75,981 utterances was therefore taken from the younger input to match the older input. CLASSIC was therefore presented with a total of 2 × 75,981 = 151,962 utterances, intended to be representative of the type of input that children receive up to the age of 6 years. Each utterance is presented to the model in full; for each utterance, CLASSIC will recode the utterance into as few chunks as possible given the chunks that are known thus far. Learning will then create a new chunk for each adjacent pair of chunks, assuming the word boundary and learning rate constraints are fulfilled.

### Parameters and assumptions in CLASSIC

CLASSIC is an intentionally parsimonious computational model that we use to illustrate how a simple associative (sequential) learning mechanism operating on the linguistic environment is able to predict human performance in language-related tasks. The model is no more complex than that described above because its goal is simply to provide an estimate of the sequential knowledge of language that a typical participant might be expected to have gained, in order to use that to predict STM performance with different types of verbal material. However, since there are certain assumptions and parameters that may influence performance of the model, we describe them here.

#### Parameter 1: learning rate of 0.50

Previous versions of CLASSIC (e.g., G. Jones, 2016) have used a learning rate of 1.00 to show how the model is able to simulate children’s performance when using a smaller input set than that outlined above. This was because the input to the model was trivial compared to the developing child, who hears up to half a million utterances in a 3-week period (Swingley, 2007). We compensate for the increase in input here by reducing the learning rate to 0.50.

#### Parameter 2: input to the model

The only other parameter relating to the current model is the input received, which is outside of the model’s architecture. Larger amounts of input lead to greater learning in the model. This has overwhelming support from language literature where a robust finding is for greater exposure to language resulting in larger vocabularies (e.g., Hoff & Naigles, 2002; Huttenlocher, Haight, Bryk, Seltzer & Lyons, 1991). Clearly, the language input is inextricably linked to the learning rate parameter; the number of chunks learned may be equivalent for a model having a large input with a small learning rate versus a small input with a large learning rate. Our goal in this regard is to show how associative learning operating on the linguistic environment is able to predict short-term memory performance, and for this we use a realistic learning rate given the paucity of language input.

#### Assumption 1: associative (sequential) learning mechanism

There is substantial support for the simple learning mechanism invoked in CLASSIC, from the recoding of familiar item sequences into larger units (e.g.., Miller, 1956) to word segmentation on the basis of statistical regularities in the input (e.g., Saffran, 2001). Within the model, every chunk learned is available to recode any subsequent utterance. No limit is placed on the learning mechanism, but since the input is one utterance at a time, the largest chunk that can be learned relates to one whole utterance (assuming sufficient exposure to that utterance). Within the memory literature, much larger feats are possible (e.g., Ericsson, Chase, & Faloon, 1980).

#### Assumption 2: word-delimited input

The model begins with only the phonemes of standard British English because we do not wish to impart any further linguistic knowledge within the model that may influence results. However, we assume that the child already knows how to segment words within continuous speech because children are already capable of determining word boundaries via a range of phonetic, phonological, and distributional cues by their first birthday (see Rowland, 2014, for a review).

#### Assumption 3: phonetic input

For the present purposes, the issue of whether phonemes are the correct basic unit (as opposed to, e.g., onsets, rimes, syllables) is not critical, since the same basic learning processes will still be at play and lead to qualitatively similar outcomes.

### Performing span and repetition tests

As discussed in the Introduction, the broad hypothesis is that performance in the vSTM setting is a function of the extent to which the material presented in that setting corresponds to the long-term linguistic knowledge of the rememberer. In operational terms here, this corresponds to the number of chunks needed to recode the test sequence: The greater the correspondence between test stimulus and long-term knowledge, the fewer chunks are required. Span and repetition test materials can therefore be presented as input to the model, and after learning we can determine how many chunks are required to recode the input, which can then be used to compare (for example) span lists involving digits versus words or nonword sets that are wordlike versus those that are not wordlike.

There is a caveat to the modeling work in that (as noted earlier) the language input is a limited reflection of the sheer amount of input that children receive. Ultimately this may have little bearing on nonword repetition results because, by definition, these are constructed from units that will only have been encountered sublexically, and exposure to the 151,962 utterances used as input means that the model is exposed to well over 2 million biphone sequences. However, the input will not reflect all of the possible digit sequences and word sequences to which the child is exposed, and, therefore, estimates involving these tests are likely to underrepresent the involvement of associative learning.

## Modeling predictions for digit span, nonword repetition, and sentence recall

### Stimuli

Measurement of digit span use is dominated by the Wechsler intelligence scales and therefore the digit span test from the Wechsler Preschool and Primary Scale of Intelligence–Third Edition, UK version (WPPSI; Wechsler, 2004) was used because the child studies involve 6-year-olds. This scale involves lists containing two to nine items, with two different digit lists at each length. Further lists were created based on the procedure used by G. Jones and Macken (2015), who investigated associative learning by comparing digit span, word span, and mixed lists that contained both digits and words. Digit lists and word lists are compared because there is substantial evidence that both children and adults are more able to recall lists containing random sequences of digits than lists containing random sequences of words (see Dempster, 1981, for a meta-analysis across child studies, and also G. Jones & Macken, 2015, for a detailed analysis concerning adults). In the current study, only nouns were considered for word lists. Each digit was matched for syllabic length and, when possible, phonemic length; however, frequency took precedence whereby nouns were selected having frequencies far greater than digits to rule out any effects that could be attributed to higher frequency for digits since they may appear as both words and numerals (see Table 2). Word lists were produced by substituting each digit in the digit span lists for its corresponding noun. Mixed lists were produced by (a) substituting each odd-numbered digit with its corresponding noun, and (b) substituting each even-numbered digit for its corresponding noun (see Table 3 for examples). The comparison of coding for matched word and digit lists in the model will allow us to examine if superior recall for digit lists over word lists can be explained by associative (sequential) learning.^1^ The mixed lists allow us to more directly examine the extent to which any differences between digits and words are due to the learning of sequential associations based on language exposure, rather than inherent characteristics of the items themselves by comparing digit recall when a digit is neither preceded nor succeeded by another digit (an “isolated” digit) versus the same for words, and digit sequence recall when a list contains a digit sequence versus a word sequence.

**Table 2:** Digit and word characteristics (frequencies taken from the Children’s Printed Word Database, Masterson, Stuart, Dixon & Lovejoy, 2010)

Digit	Phonemes/ frequency	Word match	Phonemes/ frequency
One	3/3069	House	3/1880
Two	2/1114	Water	4/1525
Three	3/706	Door	3/857
Four	3/276	School	4/1393
Five	3/173	Tree	3/995
Six	4/103	Bed	3/771
Seven	4/70	Car	2/714
Eight	2/41	Boat	3/563
Nine	3/38	Cup	3/216
	*M* = 3.0/621.1		*M* = 3.1/990.4

**Table 3:** Examples of mixed lists when odd-numbered digits are substituted for words (middle column) and when even-numbered digits are substituted for words (right column)

Example digit list	Mixed list (odd numbers substituted)	Mixed list (even numbers substituted)
Five, two, six	Tree, two, six	Five, water, bed
Six, two, nine, four	Six, two, cup, four	Bed, water, nine, school

Since young children may only be expected to recall lists containing relatively few items, two lists were changed prior to producing the mixed lists (one at List Length 3 and one at List Length 4) to ensure that all mixed lists at lengths 3 and 4 contained at least one isolated digit/word and at least one digit/word sequence. The benefit of producing two types of mixed list (one that substitutes odd numbers and one that substitutes even numbers) is that it allows direct comparison of individual digits versus individual words, and digit sequences versus word sequences, without being confounded by the serial position of the item(s) within list (see Table 3).

Two nonword repetition tests were used. The first was the Children’s Test of Nonword Repetition (CNRep; Gathercole, Willis, Baddeley, & Emslie, 1994), which uses nonwords that have been split into those that are wordlike and those that are not wordlike based on subjective ratings (Gathercole, 1995), each group containing 15 nonwords, five of each with two, three, and four syllables. However, some nonwords in both groups bear strong similarity to actual lexical items (e.g., trumpetine) or contain morphemes (e.g., tafflest; e.g., Graf Estes, Evans, & Else-Quest, 2007). We therefore added a second test from G. Jones et al. (2010), where nonwords contain no lexical items or morphemes but are split into two groups, one each having six of two, three, and four syllable nonwords containing biphones of a relatively high frequency in standard British English and the other with matched characteristics but containing significantly lower frequency biphones.

The sentence recall test was taken from the Clinical Evaluation of Language Fundamentals–Preschool–Second Edition UK (CELF-2-Preschool; Wiig, Secord, & Semel, 2006). Up to 13 sentences are read aloud by the experimenter with the child’s task being to repeat the sentences accurately. Sentences gradually increase in length from three words to 13 words.

For all stimuli, the relevant item or list was presented to the model and was recoded in as few chunks as possible, with the number of chunks being recorded. For example, a list of digits would be given as input to the model, and the number of chunks required to recode the list would be recorded.

### Model predictions

Note that for all of the modeling results, there is no variability in the model’s performance—all figures represent the actual number of chunks required to recode the respective lists, and therefore any differences are real differences in the model’s performance. Number of chunks, therefore, provides a simple estimate of processing efficiency due to prior learning (e.g., Ericsson et al., 1980; Miller, 1956).

### Digit span, word span, and mixed span

**Fig. 1:**
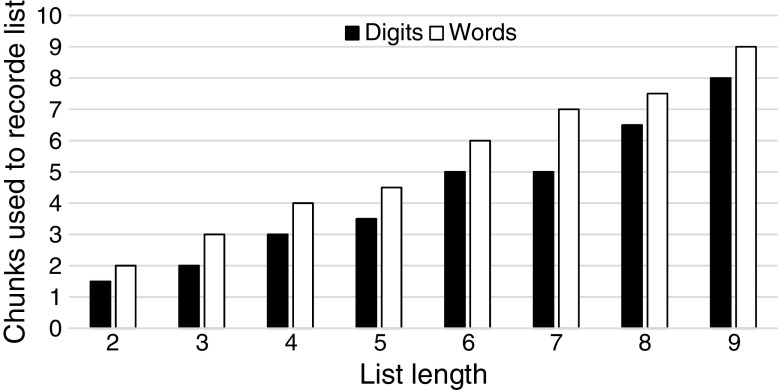
Chunks used to recode digit lists and word lists, for all possible list lengths

**Fig. 2:**
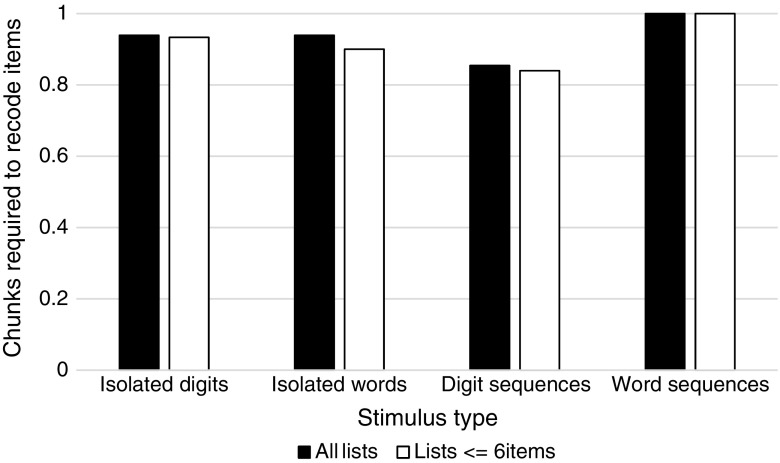
Average number of chunks used to recode isolated digits, isolated words, digit sequences, and word sequences, for all lists and for lists containing six items or fewer

We first examine the number of chunks required to recode digit lists and word lists, shown in Fig. 1. At all list lengths, fewer chunks are required to recode lists containing digits than lists containing words, suggesting that an associative learning account operating on language exposure predicts superior performance for digit span over word span. For mixed lists, we separately examine all lists and lists up to a length of six items, on the assumption that 6-year-old children are unlikely to proceed further than six items on such a test. Figure 2 shows the number of chunks that are required to recode isolated digits, isolated words, digit sequences, and word sequences. When considering all lists, there is no difference in the number of chunks used to recode isolated digits and isolated words, but a marked difference appears when considering sequences where digit sequences are recoded into fewer chunks than word sequences. When considering list lengths up to six items, a similar pattern appears, though there are marginally fewer chunks required to recode isolated words (this is because on occasion a digit-word or word-digit sequence forms a phonetic chunk, e.g., *to bed* [two-bed]). Based on associative learning, one may expect children to recall digit sequences more accurately than word sequences but perform similarly for isolated digits and words.

### Nonword repetition

The model is exposed to well over 2 million biphone sequences and only requires two occurrences of a sequence to learn it. For nonwords, this presents a stiff test of associative learning by acting against our hypothesis because it means that even low-frequency biphone sequences may be learnt quickly, potentially limiting any difference between low-frequency and high-frequency sequences. Table 4 shows the number of chunks required to recode wordlike versus nonwordlike nonwords and low versus high phonotactic probability nonwords. Wordlike nonwords are consistently recoded using fewer chunks than nonwordlike nonwords for all nonword lengths, despite wordlike nonwords being phonemically longer items than nonwordlike nonwords and despite the model learning at every other opportunity (i.e., a learning rate of .50). Similarly, high phonotactic probability nonwords are recoded using fewer chunks than low phonotactic probability nonwords. An associative learning account therefore suggests that repetition differences will arise across nonword sets purely on the basis of the child’s experience of the sequences that comprise the nonwords. Furthermore, it is notable that even low phonotactic probability nonwords that do not contain any morphemes benefit substantially from associative learning, with the effective length (i.e., number of chunks required to recode nonwords) being reduced by more than 38%.

**Table 4:** Average number of chunks required to recode wordlike (WL), nonwordlike (NWL), high phonotactic probability (HPP), and low phonotactic probability (LPP) nonwords at each syllabic length

	2 syllable	3 syllable	4 syllable
WL, phonemic length	5.60	7.80	10.30
WL, recoded chunks	2.20	2.80	3.80
NWL, phonemic length	5.00	7.00	9.00
NWL, recoded chunks	2.83	3.83	4.83
HPP, phonemic length	5.00	7.00	9.00
HPP, recoded chunks	2.33	3.33	4.33
LPP, phonemic length	5.00	7.00	9.00
LPP, recoded chunks	3.33	4.33	5.33

### Sentence recall

The average length (in words) of the sentences used for sentence recall is 8.54. These sentences are recoded using an average of 5.96 chunks, suggesting that sentence recall is supported by associative learning of the word sequences involved. One can also examine the possibility of differences in associative learning capability by altering the learning rate from .50 to .10 (i.e., requiring on average 10 instances of a sequence in order to learn it). This increases the average number of recoded chunks to 7.38, suggesting that differences in the amount of language knowledge learned (arising from differences in associative learning) are likely to cause significant differences in sentence recall ability. This is something we will return to when examining the sentence recall data from the behavioral studies.

We should note that altering the learning rate also changes the performance of the model for other stimuli. For example, the chunks needed to recode high phonotactic probability nonwords increase from an average of 3.33 to an average of 3.93, while those required to recode low phonotactic nonwords increase from 4.33 to 5.33. Although not the focus of this article, this does show how individual differences can be captured within CLASSIC, in this case, showing how reductions in learning rate have more impact on low phonotactic probability nonwords as opposed to high phonotactic probability nonwords (the same effects are seen when comparing typically developing children and children with specific language impairment; G. Jones et al., 2010).

### Modeling summary

For every test presented, a simple associative learning account has indicated that substantial enhancements in the coding of novel sequences is possible due to the sequential characteristics of the information within the tests, combined with the content of the natural linguistic environment of the child. We now turn to behavioral studies involving 6-year-old children to determine whether these predictions correspond to children’s performance.

## Behavioral experiments: overview

We measured the performance of two groups of 6-year-old children on the same stimuli presented for encoding to the model. Experiment 1 tested recall of digit and word lists, and Experiment 2 tested nonword repetition and sentence recall. In addition, in Experiment 2, we assessed individual participant’s language knowledge using the core language subtests in the CELF-2-Preschool in order to examine the relationship between language knowledge and performance on the repetition tasks on an individual differences basis. The broad objective of the behavioral studies was to examine the extent to which encoding efficiency (i.e., number of chunks required to recode input), attributable solely to domain-general associative learning processes operating on the child’s linguistic environment, could predict the pattern of vSTM performance seen in our sample of children. While the issues addressed in Experiment 2 are not completely novel (e.g., effects of wordlikeness have already been shown for nonwords), we include this study because the model predictions are based on specific nonword sets and a specific sentence recall test, and therefore Experiment 2 will allow a test of the model on specific sets of stimuli rather than merely ones defined by broad linguistic characteristics.

## Experiment 1: children’s digit span, word span, and mixed span

### Design

For digit and word lists, the independent variable was stimulus type (digits or words), and the dependent variable was the number of lists correctly recalled. For mixed lists, since sequences could involve more than two items (e.g., *three-eight-two*), each paired sequence was examined (i.e., 1 point for correct recall of *three-eight* and 1 point for *eight-two*). The independent variables were therefore stimulus type (digits or words) and sequence type (isolated item or item pair), with the dependent variable being the number of correct recalls of the relevant item (e.g., digit pair).

### Participants

Thirty 6-year-olds (*M* = 75.33 months, *SD* = 3.69, range: 70–81 months, 14 female) were recruited from schools within the Nottinghamshire, UK, area. All children spoke English as their first language. As with all experiments reported here, participants were treated in accordance with British Psychological Society ethical principles, and the research received ethical approval from the Nottingham Trent University Social Sciences ethics committee.

### Materials

The digit span, word span, and mixed lists outlined in the model predictions section were used as stimuli. Each item was recorded individually using Audacity (e.g., the spoken form of the digits 1 through 9 were recorded individually). Digit lists, word lists, and mixed lists were then created as MP3 files by constructing an individual sound file for each list, with each successive item being separated from its predecessor by .7 seconds of silence.

### Procedure

Each child was tested outside of the class environment and in a quiet area of the school. For all types of lists, testing began at the shortest list length. Both lists at a particular list length were presented using a Sony ICD-MX20 digital voice recorder, with list length increasing only when at least one of the lists was accurately recalled (this is the test method for digit span within the WPPSI). The child’s task was to verbally recall each list immediately after presentation of the list. List presentation was counterbalanced, and testing sessions normally lasted approximately 10 minutes. The same researcher carried out all testing.

### Results

The number of digit lists correctly recalled was 6.23 (CI 5.73, 6.74) and the number of word lists accurately recalled was 4.97 (CI = 4.38, 5.55). As expected from the associative learning account described, there were significantly more digit lists recalled than word lists, *t*(29) = 6.24, *p* < .001, Cohen’s *d* = .86. Table 5 shows the data for the mixed lists. There was a significant effect of sequence type, with isolated items being recalled more accurately than item pairs, *F*(1, 29) = 224.06, *p* < .001, η_p_^2^ = .89, but no effect of stimulus type, *F*(1, 29) = 1.13, *p* = .297, η_p_^2^ = .04. However, there was an interaction between sequence type and stimulus type, *F*(1, 29) = 13.10, *p* = .001, η_p_^2^ = .31, illustrating that isolated words were recalled more accurately than isolated digits, yet digit pairs were recalled more accurately than word pairs, with Bonferroni adjustment, *t*(29) = 3.27, *p* = .006, Cohen’s *d* = .23 for isolated items; *t*(29) = 2.89, *p* = .014, Cohen’s *d* = .53, for item pairs. The model showed a slight advantage for isolated words over isolated digits only for lists of six items or fewer (see Fig. 2). Nevertheless, the superior recall of isolated words over digits in the child data supports our general hypothesis that superior performance for digit lists over word lists arises due to the greater experience with digit sequences, rather than being due to any inherent characteristics of digits as items, because in isolation they do not support superior short-term memory. The effect of associative learning across digit sequences must be considerable because it reverses the pattern of performance found here when memory for individual items is assessed, leading to the typical finding of superior recall for digit lists over word lists.

**Table 5:** Means and confidence intervals (in parentheses) for the different conditions of the mixed list stimuli

Sequence type	Stimulus type	
	Digits	Words
Isolated item	6.40 (5.82–6.98)	6.77 (6.16–7.38)
Item pair	4.63 (4.26–5.01)	4.07 (3.65–4.48)

## Experiment 2: children’s nonword repetition and sentence recall

Although linguistic influences on nonword repetition and sentence recall have previously been shown (e.g., Archibald & Joanisse, 2009; G. Jones et al., 2010), Experiment 2 enables us to examine whether the predictions of the model hold for the specific stimuli that were applied to the modeling environment.

### Design

For wordlikeness effects in nonword repetition, the independent variables were nonword type (wordlike or nonwordlike) and nonword length (two, three, or four syllables). For phonotactic probability effects, the independent variables were nonword type (high phonotactic probability or low phonotactic probability) and nonword length (two, three, or four syllables). In both cases, the dependent variable was the number of nonwords accurately repeated. For sentence recall, the independent variable was language ability (low or high) and the dependent variable was a score based on the CELF-2-Preschool scoring procedure: A score of 3 is given when a sentence is repeated accurately, a score of 2 is given when one morpheme is omitted, a score of 1 is given when two or three morphemes are omitted, and a score of 0 is given otherwise. The minimum score on this test is therefore 0, with a maximum score of 39.

### Participants

Thirty-four 6-year-olds (*M* = 73.06 months, *SD* = 5.74, range: 62–85 months, 16 female) were recruited from schools within the Nottinghamshire area. Note these children were not the same as those used in Experiment 1. All children spoke English as their first language.

### Materials

The nonword repetition tests were the same as those used in the model simulation and were recorded onto a Sony ICD-MX20 digital voice recorder. Due to their length, the tests were split into smaller lists, with presentation order counterbalanced. Sentence recall was administered from the sentence recall task of the CELF-2-Preschool, along with the three core tests of language ability (sentence structure, word structure, and expressive vocabulary) from the same test so that we had a measure of the general language ability of each child (this was not done in Experiment 1).

### Procedure

Each child was tested outside of the class environment and in a quiet area of the school. Repetition tests were administered along with sentence recall and the three core language tests. Testing was normally spread across three testing sessions, with presentation order of the experimental materials and standardized tests being counterbalanced and each testing session lasting approximately 10 to 15 minutes. The same researcher carried out all testing.

### Results

Table 6 shows the nonword repetition results for both nonword tests. For wordlikeness, there was a significant effect of nonword type, *F*(1, 33) = 7.82, *p* = .009, η_p_^2^ = .19, with repetition accuracy being greater for wordlike than for nonwordlike nonwords. There was an effect of nonword length, *F*(2, 66) = 204.88, *p* < .001, η_p_^2^ = .86, indicating that short nonwords were repeated more accurately than long nonwords. There was no interaction between nonword type and nonword length, *F*(2, 66) = 1.75, *p* = .182, η_p_^2^ = .05. The phonotactic probability manipulation follows the same pattern, with effects of nonword type, *F*(1, 33) = 6.49, *p* = .016, η_p_^2^ = .16, indicating superior repetition accuracy for high over low phonotactic probability nonwords; and nonword length *F*(2, 66) = 95.76, *p* < .001, η_p_^2^ = .74, indicating a decline in repetition accuracy as length increases. There was no interaction between the two, *F*(2, 66) = .09, *p* = .913, η_p_^2^ = .01. On the one hand, these results are not surprising, because wordlikeness and phonotactic probability effects have previously been demonstrated for these tests (e.g., G. Jones et al., 2010). However, because the model simulation presented above shows exactly the same pattern based solely on associative (sequential) learning processes operating on the linguistic environment, the need to invoke specific short-term memory processes to account for the different performance with different types of verbal material and to account for the developmental changes in that performance is obviated. Moreover, associative learning accounts not only for differences across nonword sets but also for performance differences across nonword lengths. This indicates that differences in performance in the vSTM setting may be accounted for by reference to the extent to which the linguistic repertoire of the participant corresponds to the type of material presented in that short-term setting without having to invoke a limited capacity STM system per se. A key aspect of that repertoire depends on domain-general associative learning processes operating on the linguistic environment.

**Table 6:** Means and confidence intervals (in parentheses) for wordlike (WL), nonwordlike (NWL), high phonotactic probability (HPP), and low phonotactic probability (LPP) repetition tests

	Nonword length		
	2 syllable	3 syllable	4 syllable
WL	62.06 (56.51–67.61)	41.76 (36.95–46.58)	25.88 (19.74–32.02)
NWL	54.17 (46.32–62.02)	37.75 (28.91–46.58)	13.73 (8.42–19.03)
HPP	57.84 (48.43–67.26)	40.69 (30.96–50.42)	16.18 (9.70–22.66)
LPP	50.49 (41.05–59.93)	34.80 (25.16–44.44)	11.27 (5.60–16.95)

To further investigate the role of associative learning, we examined repetition performance on a nonword-by-nonword basis, analyzing the relationship between children’s performance for individual nonwords and the number of chunks required to recode those same nonwords. There was a significant correlation between the two, *r*(64) = -.63, *p* < .001. This was also the case when examining by nonword set, *r*(28) = −.55, *p* = .002, and *r*(34) = −.72, *p* < .001, for the wordlikeness and phonotactic probability sets respectively).

To examine the relationship between language function and sentence recall, a median split was carried out on the CELF core language scores that combine the sentence structure, word structure, and expressive vocabulary subtests in order to separate children into groups: those with a large amount of language knowledge (CELF scores > 96) versus those with a relatively small amount of language knowledge (CELF scores <= 96). The rationale here is that the CELF scores provide an indication of linguistic knowledge and experience at the level of the individual (in the model, we manipulated language experience by altering the learning rate). Perhaps unsurprisingly, though nonetheless in line with the model’s predictions, sentence recall was significantly better for the children having high CELF scores (29.87) than it was for the children having low CELF scores (18.75), *t*(29) = 5.28, *p* < .001, Cohen’s *d* = .69.

If, as we are arguing, the pattern of performance for nonword repetition and sentence recall is attributable to long-term learning operating on the linguistic environment of the child rather than being driven by the development of basic, bespoke short-term memory systems, then we would predict that the child’s level of linguistic knowledge will show a stronger relationship to repetition abilities than maturational factors, such as age, that are often linked to increases in vSTM capacity (e.g., Baddeley et al., 1998). Table 7 shows correlations across age, CELF scores, the two repetition tests, and sentence recall.^2^ As the table demonstrates, age has little relation to repetition ability, whereas language ability is strongly related to all aspects of repetition.

**Table 7:** Correlations between age, CELF, wordlikeness (WL), and phonotactic probability (PP) manipulations of nonword repetition test, and sentence recall

	CELF	WL nonwords	NWL nonwords	Sentence recall
Age	.27	.24	.22	.00
CELF		.42*	.50**	.67**
WL nonwords			.50**	.45**
PP nonwords				.56**

*p < .05. **p < .01

### Child-model summary

The model suggests that associative learning plays a role in children’s performance on digit span, nonword repetition, and sentence recall by increasing the efficiency with which the child processes the verbal strings presented to them in vSTM tasks due to their linguistic experience. Table 8 shows the relative difference in recoded chunks across the different conditions within the vSTM tasks, together with the relative difference in children’s performance on the same tasks. The size of the influence of experience is clearly similar in both the model and the children for all but the sentence recall task, where linguistically experienced children show substantially greater improvement than that shown in the model. This is perhaps unsurprising, given that the model only learns pairwise associations, while sentence recall in children is likely to reflect more than simple sequence learning (e.g., aspects of knowledge such as semantic and syntactic transitional probabilities). We return to this issue in the General Discussion.

**Table 8:** Performance differences across different stimuli sets, for the model and the children

	Recoded chunks	Chunk differential	Children’s performance	Performance differential
Digit span vs. word span	4.31 vs. 5.38	20%	6.23 vs. 4.97	20%
Isolated digits vs. isolated words	31.00 vs. 31.00	0%	6.40 vs. 6.77	5%
Digit sequences vs. word sequences	47.00 vs. 55.00	15%	4.63 vs. 4.07	12%
WL nonwords vs. NWL nonwords	2.93 vs. 3.83	23%	43.23 vs. 35.21	19%
HPP nonwords vs. LPP nonwords	3.33 vs. 4.33	23%	38.23 vs. 32.19	16%
Sentence recall, high CELF vs. low CELF	5.96 vs. 7.38	19%	29.87 vs. 18.75	37%

WL = wordlike; NWL = nonwordlike; HPP = high phonotactic probability; LPP = low phonotactic probability

Overall, the pattern of performance of 6-year-olds in the range of vSTM tasks assessed here mirrors that of the model simulation of the amount of sequential associative learning that may take place due to exposure to the linguistic environment of the child. At both the level of different types of test stimuli (digit and word lists, nonwords, sentences) and individual items (nonwords), vSTM performance in the children corresponded to the efficiency with which the model encoded those stimuli. Furthermore, while the relationship between the child’s age and his or her performance on the repetition tasks was weak and nonsignificant, that performance was positively related to language proficiency, as measured by CELF scores.

## General discussion

We set out to examine whether patterns of vSTM performance in children found with different types of verbal material could be accounted for by reference to simple domain-general associative processes operating on the linguistic experience of the child. CLASSIC used a simple sequential learning procedure to estimate the kind of associative learning that may take place for linguistic stimuli. Our model simulation began with a repertoire whose units were restricted to basic British English phonemes and was then presented with corpora representing the linguistic experience of a 6-year-old. Word-delimited utterances in the corpora were encoded using the fewest possible units in the available repertoire, and learning took place via the formation of new units (chunks) for pairs of adjacent units in the input. After training on the corpora, the model was then tested with the types of stimuli typically used in tests of vSTM in children, revealing increased efficiency (fewer chunks) in the encoding of digits versus word sequences, of wordlike and phonotactically regular versus nonwordlike and phonotactically irregular nonwords, as well as showing experience-based improvements in encoding of novel sentences. The performance of 6-year-old children for tests relating to digit span, word span, mixed span, and nonword repetition in Experiments 1 and 2 mirrored these processing efficiencies, down to the level of short-term recall of individual nonwords, as well as revealing a relationship between linguistic experience and sentence recall in both model and child. Furthermore, the magnitude of performance differences across different stimuli sets was similar between model and child for all stimuli except sentence recall. The pattern of performance, then, implicates basic associative learning processes operating on the child’s experience with language as underpinning his or her performance in the typical short-term memory tasks used in developmental studies seeking to determine the role of basic short-term memory processes in the development of higher level cognitive functions. This work shows that performance on widely used short-term memory tasks can be predicted by reference to associative learning mechanisms that are known to be involved even in infancy (e.g., Saffran, 2001). In short, our computational model of associative learning provides a parsimonious explanation of performance in vSTM tasks without the need for additional bespoke processes such as a short-term memory system. Rather than being viewed as a specific processing system, we suggest that vSTM be viewed as a particular setting in which the participant applies his or her language knowledge to the task at hand.

It is worth noting that the model presented here successfully predicts the short-term memory performance of the children while implementing a very limited (unrealistically so) set of constraints with respect to its knowledge base and the type of learning that occurs. So, the linguistic repertoire used to encode the input is the inventory of English phonemes, while, for any given point on the developmental trajectory, a more realistic representation would plausibly include some combination of subphonemic knowledge (such as acoustic/articulatory features, as well as larger units, such as onsets, rimes, syllables, and even longer multisegment strings, knowledge of which may precede knowledge of smaller segments, e.g., Bybee, 2010; Vihman, 2014). Similarly, while the model only builds up knowledge based on adjacent pairwise co-occurrences in the input, longer range dependencies and predictive relationships are also a critical aspect of natural language learning, and so a more realistic reflection of the learning that can be accomplished from the input would also have to incorporate such processes. We suggest that the limited nature of the learning that takes place in the model underlies its lesser ability to capture performance on sentence recall—a task likely to benefit more from just such longer range semantic and syntactic learning—compared to the other vSTM tasks. However, given that the rarefied implementation presented here nonetheless is able to mirror children’s performance on vSTM tasks, it is not unreasonable to suggest that a more realistic implementation, containing a more refined and elaborate set of knowledge units and associative learning processes, would likely provide a more powerful model of performance on short-term memory tests, without having to invoke short-term memory processes per se. Also, our findings are not restricted to the particular stimuli presented in the vSTM measures used. Similar effects occur when different sets of word span lists are used to compare digit span in adults (G. Jones & Macken, 2015), the number of chunks required to recode an array of nonword lists correspond to children’s repetition performance for the same nonwords (G. Jones, 2016), and sentence recall is influenced by associative learning for 200 novel sentences (G. Jones & Rowland, 2017).

Our findings contribute to a growing body of evidence that implicates domain-general long-term learning processes in performance on vSTM tasks, something that in itself is not novel (e.g., Botvinick, 2005; Botvinick & Plaut, 2006; Majerus et al., 2012), although its theoretical consequences remain controversial. The close correspondence between performance in the vSTM setting and the participant’s linguistic skill and knowledge (see, e.g., G. Jones & Macken, 2015, and B. Macken et al., 2014, for discussion) points, we suggest, to a view of vSTM not as a set of systems or processes in itself but rather as a setting within which the participant must flexibly bring to bear his or her knowledge and skill in order to accomplish the goals in what is, by design, a (relatively) novel task involving a (relatively) novel set of materials. For vSTM, this means that the task differs only in operational terms from other types of novel verbal settings, such as those examined in typical psycholinguistic experiments where people are required to deal with, for example, complex syntactic structures (e.g., Farmer, Fine, Misyak, & Christiansen, 2016) or find productive ways of dealing with lexically novel items (e.g., Bybee, 2010). Although the functioning of vSTM processes have often been invoked to explain performance in such psycholinguistic settings, the weight of evidence here increasingly points to the nature and extent of linguistic experience and how closely it corresponds to the task setting (e.g., Farmer et al., 2016; Frank, Tromenaars, & Vasishth, 2015).

We have shown in principle that vSTM performance can be modeled without invoking the type of capacity-limited process that is assumed under most accounts of short-term memory (e.g., Baddeley et al., 1998; Jefferies, Frankish, & Noble, 2009). Instead, the limits to vSTM performance arise from two broad principles, one being the opportunity for long-term learning about the structure of language and the other being the degree of correspondence between that long-term knowledge and the particular task and set of materials presented to the participant in the vSTM setting. What appears as “capacity limitation” in this setting is instead a specific performance limitation arising from the mismatch between the task setting and the actual environment from which the rememberer has acquired his or her linguistic knowledge (B. Macken et al., 2016).

Although our approach does not invoke a capacity-limited STM system to explain performance in the STM setting, neither does it require it to be a system for the initial learning of language knowledge, as is often the case in theorizing about the relationship between vSTM and language (Baddeley et al., 1998; Page & Norris, 2009). The latter approach makes the assumption that because relevant information exists at a short-term temporal scale, there must be a short-term memory system to enable learning about that (i.e., the classical view that STM serves as a gateway to long-term learning; Atkinson & Shiffrin, 1968). However, if we consider the knowledge possessed by a skilled user of language in the broadest sense (i.e., the ability to perceive and produce language), then it is clear that relevant knowledge exists across a wide range of temporal scales—from the simultaneous occurrence of formant relations, to the few milliseconds over which other phonetic contrasts are discriminated, to the tens and hundreds of milliseconds over which phonotactic, morphemic, syllabic, and lexical knowledge is represented, through the several seconds over which syntactic and utterance-level information occurs, through to the minutes and hours whereby the structure of conversations may be discerned. Knowledge of language involves learning about structure at all these scales, and it seems to us implausible and unparsimonious to propose a bespoke system for knowledge at one of these levels of scale (i.e., whatever scale might be conceived of as “short-term”), rather than proposing that domain-general statistical learning processes operate across time scales.

In eschewing the concept of short-term memory as a cognitive system in itself, such an orientation raises the possibility that questions about the variety of empirical relationships between short-term memory performance and higher cognitive functions, particularly with respect to the development of those functions, should be reframed. Indeed, the implication is that short-term memory, rather than being a concept connoting a key component of cognitive processing, is a reification of the particular types of task characteristics that are used to measure performance, particularly those related to the processing of material that is novel with respect to the rememberer’s experience. As we have shown here, this novelty is always a matter of degree, and rather than seeking to partition bespoke short-term memory processes from other, long-term learning processes, our argument is that it is precisely these long-term learning processes, and the ability to flexibly co-opt the knowledge and skill so acquired, that are under investigation in the short-term memory setting. From this perspective, rather than short-term memory playing a causal role in the development of higher order cognitive functions, it is instead an outcome of that development.

## Footnotes

1 Note that we do not consider the possibility that known digit pairs may interfere with to-be-remembered digit pairs. This is because children’s learning of frequently encountered word combinations only serve to facilitate performance on language-related tasks (e.g., Bannard & Matthews, 2008), with no evidence to suggest that known word combinations interfere with that performance.

2 Although we cannot rule out the possibility that the failure to find significant correlations with age may be due to insufficient power, it is nonetheless clear that there is sufficient power with *N* = 34 to detect the other correlations, and it can at least be concluded that the role of age is considerably less than that of the other factors.
